# Draft Genome Sequence of *Pseudomonas veronii* Strain 1YdBTEX2

**DOI:** 10.1128/genomeA.00258-13

**Published:** 2013-05-16

**Authors:** Daiana de Lima-Morales, Diego Chaves-Moreno, Michael Jarek, Ramiro Vilchez-Vargas, Ruy Jauregui, Dietmar H. Pieper

**Affiliations:** Microbial Interactions and Processes Research Group, Helmholtz Centre for Infection Research, Braunschweig, Germanya;; Genome Analytics, Helmholtz Centre for Infection Research, Braunschweig, Germanyb

## Abstract

*Pseudomonas veronii* strain 1YdBTEX2 was isolated from a benzene-contaminated site. Here we report the draft genome sequence of 1YdBTEX2 and its genes associated with aromatic metabolism. The broad catabolic potential of this strain is consistent with the environment from which it was isolated.

## GENOME ANNOUNCEMENT

Environmental spills with recalcitrant pollutants are a common problem worldwide, threatening the environment and human health. The main mechanism for recovering these sites is through biodegradation by indigenous microorganisms. The reasons for some microorganisms persisting and successfully establishing under pollutant pressure are still to be elucidated.

Here we present the draft genome of *Pseudomonas veronii* strain 1YdBTEX2, isolated from soil highly contaminated with benzene (1). This strain harbors a unique catabolic pathway for the degradation of both benzene and toluene and has been purported to be a key player in the biodegradation of these pollutants at this site (1, 2). While the 16S rRNA gene of 1YdBTEX2 is 99.6% similar to that of *P. veronii* CIP104663^T^ and 98.7% similar to that of *Pseudomonas fluorescens* IAM12022^T^, the housekeeping gene *gyrB* is 99.2% similar to that of *P. veronii* CIP104663^T^ and only 91.0% similar to that of *P. fluorescens* IAM12022^T^ (http://www.uib.es/microbiologiaBD/Welcome.html). Thus, 1YdBTEX2 is the first member of the *P. veronii* species to be sequenced.

The genome was sequenced using the Illumina GAII genome analyzer, which generated 110-bp paired-end reads. Approximately 9.5 million reads were obtained and the whole genome was assembled into 281 contigs using Velvet (3) and 344 contigs using Edena (4). Both datasets were then merged using Minimus2 (5), resulting in 119 contigs. In order to merge contigs, we constructed scaffolds using SSPACE (6). Comparisons between contigs and scaffolds were then made using Mauve (7), using the genome of *P. fluorescens* SBW25 (8) as a reference. Gaps between scaffolds were closed by PCR amplification followed by DNA Sanger sequencing. Annotation was performed with the RAST (Rapid Annotations using Subsystems Technology) server version 4.0 (9), generating 5,981 candidate protein-encoding genes. The resulting genome sequence consists of 63 contigs (N_50_ = 229,690; L_50_ = 10) of 6,680,724 bp with a GC content of 59%. Besides the peripheral aromatic degradation pathways for benzene/toluene, phenol, salicylate, tryptophan (via anthranilate), ferulate (via vanillate), p-coumarate (via 4-hydroxybenzoate), 3-hydroxyphenylpropionate, and phenylalanine (via 4-hydroxyphenylpyruvate), central aromatic degradation pathways for catechol (both via intra- and extradiol cleavage), protocatechuate (via intradiol cleavage), homogentisate, and 2,3-dihydroxyphenylpropionate and an *alkB* gene encoding alkane monooxygenase were also observed in strain 1YdBTEX2. Furthermore, while 1YdBTEX2 is versatile with respect to its use of a range of carbon sources, a cluster of genes comprising a complete denitrification pathway, including nitrate, nitrite, nitric oxide, and nitrous oxide reduction, was found, conferring flexibility in periods of anoxia. The presence of a (NiFe) hydrogenase with an entire operon similar to that found in *Ralstonia eutropha* H16 (10) was identified here, which indicates that hydrogen may be used as an electron donor, a feature unknown in *Pseudomonas* strains, with the exception of *P. extremaustralis* (11). The data are consistent with the environment from which 1YdBTEX2 was isolated and underline its broad catabolic potential.

### Nucleotide sequence accession numbers.

This whole-genome shotgun project has been deposited at DDBJ/EMBL/GenBank under the accession number AOUH00000000. The version described in this paper is the first version, AOUH01000000.

## References

[1] Junca H Pieper DH 2004 Functional gene diversity analysis in BTEX contaminated soils by means of PCR-SSCP DNA fingerprinting: comparative diversity assessment against bacterial isolates and PCR-DNA clone libraries. Environ. Microbiol. 6:95–110 14756875 10.1046/j.1462-2920.2003.00541.x

[2] Witzig R Junca H Hecht HJ Pieper DH 2006 Assessment of toluene/biphenyl dioxygenase gene diversity in benzene-polluted soils: links between benzene biodegradation and genes similar to those encoding isopropylbenzene dioxygenases. Appl. Environ. Microbiol. 72:3504–3514 16672497 10.1128/AEM.72.5.3504-3514.2006 PMC1472391

[3] Zerbino DR Birney E 2008 Velvet: algorithms for de novo short read assembly using de Bruijn graphs. Genome Res. 18:821–829 18349386 10.1101/gr.074492.107 PMC2336801

[4] Hernandez D François P Farinelli L Osterås M Schrenzel J 2008 De novo bacterial genome sequencing: millions of very short reads assembled on a desktop computer. Genome Res. 18:802–809 18332092 10.1101/gr.072033.107 PMC2336802

[5] Sommer DD Delcher AL Salzberg SL Pop M 2007 Minimus: a fast, lightweight genome assembler. BMC Bioinformatics 8:64 17324286 10.1186/1471-2105-8-64 PMC1821043

[6] Boetzer M Henkel CV Jansen HJ Butler D Pirovano W 2011 Scaffolding pre-assembled contigs using SSPACE. Bioinformatics 27:578–579 21149342 10.1093/bioinformatics/btq683

[7] Rissman AI Mau B Biehl BS Darling AE Glasner JD Perna NT 2009 Reordering contigs of draft genomes using the mauve aligner. Bioinformatics 25:2071–2073 19515959 10.1093/bioinformatics/btp356 PMC2723005

[8] Silby MW Cerdeño-Tárraga AM Vernikos GS Giddens SR Jackson RW Preston GM Zhang XX Moon CD Gehrig SM Godfrey SA Knight CG Malone JG Robinson Z Spiers AJ Harris S Challis GL Yaxley AM Harris D Seeger K Murphy L Rutter S Squares R Quail MA Saunders E Mavromatis K Brettin TS Bentley SD Hothersall J Stephens E Thomas CM Parkhill J Levy SB Rainey PB Thomson NR 2009 Genomic and genetic analyses of diversity and plant interactions of *Pseudomonas fluorescens*. Genome Biol. 10:R51 19432983 10.1186/gb-2009-10-5-r51 PMC2718517

[9] Aziz RK Bartels D Best AA DeJongh M Disz T Edwards RA Formsma K Gerdes S Glass EM Kubal M Meyer F Olsen GJ Olson R Osterman AL Overbeek RA McNeil LK Paarmann D Paczian T Parrello B Pusch GD Reich C Stevens R Vassieva O Vonstein V Wilke A Zagnitko O 2008 The RAST server: rapid annotations using subsystems technology. BMC Genomics 9:75 18261238 10.1186/1471-2164-9-75 PMC2265698

[10] Burgdorf T van der Linden E Bernhard M Yin QY Back JW Hartog AF Muijsers AO de Koster CG Albracht SP Friedrich B 2005 The soluble NAD+-reducing [NiFe] hydrogenase from *Ralstonia eutropha* H16 consists of six subunits and can be specifically activated by NADPH. J. Bacteriol. 187:3122–3132 15838039 10.1128/JB.187.9.3122-3132.2005 PMC1082810

[11] Tribelli PM Raiger Iustman LJ Catone MV Di Martino C Revale S Méndez BS López NI 2012 Genome sequence of the polyhydroxybutyrate producer *Pseudomonas extremaustralis*, a highly stress-resistant Antarctic bacterium. J. Bacteriol. 194:2381–2382 22493195 10.1128/JB.00172-12 PMC3347083

